# Chemometrics Approaches in Forced Degradation Studies of Pharmaceutical Drugs

**DOI:** 10.3390/molecules24203804

**Published:** 2019-10-22

**Authors:** Benedito Roberto de Alvarenga Junior, Renato Lajarim Carneiro

**Affiliations:** Department of Chemistry, Federal University of São Carlos, São Carlos 13565-905, Brazil; benedito.alvarenga@outlook.com

**Keywords:** forced degradation, degradation products, stress test, chemometrics

## Abstract

Chemometrics is the chemistry field responsible for planning and extracting the maximum of information of experiments from chemical data using mathematical tools (linear algebra, statistics, and so on). Active pharmaceutical ingredients (APIs) can form impurities when exposed to excipients or environmental variables such as light, high temperatures, acidic or basic conditions, humidity, and oxidative environment. By considering that these impurities can affect the safety and efficacy of the drug product, it is necessary to know how these impurities are yielded and to establish the pathway of their formation. In this context, forced degradation studies of pharmaceutical drugs have been used for the characterization of physicochemical stability of APIs. These studies are also essential in the validation of analytical methodologies, in order to prove the selectivity of methods for the API and its impurities and to create strategies to avoid the formation of degradation products. This review aims to demonstrate how forced degradation studies have been actually performed and the applications of chemometric tools in related studies. Some papers are going to be discussed to exemplify the chemometric applications in forced degradation studies.

## 1. Chemometrics

The Swedish word “kemometri” appeared for the first time in 1971 by a combination between the terms chemistry and -metri. In 1972, the English homologous term chemometrics (chemo + metrics) was referred by Prof. Svante Wold that named his group as Forskningsgruppen för Kemometri (Research Group for Chemometrics) or Kemometrigruppen (Chemometrics Group), and in the next year, it was published the first article with the term kemometri [1,2]. The International Chemometrics Society explained the term “chemometrics” for the first time in 1974. International journals, in the 1980s, had special issues on chemometrics. In 1986–1987, the publishers Wiley and Elsevier created the chemometrics journals “The Journal of Chemometrics” and “Chemometrics and Intelligent Laboratory Systems,” respectively [3].

The definition of chemometrics is intimately linked to what it is expected to gain from using it. This definition has presented some inconsistencies between authors over the years, once each one belongs to fields with different aims [4].

According to Pure and Applied Chemistry (IUPAC), the full definition of chemometrics, considering no preference of area, is the science of relating measurements performed on a chemical system or process to the state of the system through application of mathematical or statistical methods. IUPAC also highlights that, in chemometrics, the data are treated commonly in a multivariate approach, and although there are cases in theoretical chemistry that use the same mathematical and statistical techniques in some application, it should aim primarily to extract useful chemical information of measured data [5].

This definition evidences clearly the utilization of chemometrics in all stages of the chemical measurement process, from definition of optimal experimental conditions, data collection, and processing of data. Chemometrics has its roots in analytical chemistry [6], but it is totally interdisciplinary and has been applied in many different areas [7], such as food sciences [8,9,10,11,12], assessment of adulteration, geographical origin [13,14,15], metabolomics [16,17,18], engineering [19,20], forensics [21,22,23,24,25], pharmaceutical studies [26,27,28,29,30], cultural studies [31,32,33], environmental chemistry [34], etc. Chemometric tools are fundamental to solve real life problems [35].

In fact, when chemometric is applied appropriately with suitable interpretations, it enables to obtain a better data visualization even from experimental of poor quality (low resolution and high level of noise), making the relations between analytical signals and experimental parameters clearer [36]. The development of methods for analysis of degradation products is a hard work, time consuming, and an expensive task. In this context, chemometric tools are an alternative approach to carry out studies related to impurities in pharmaceutical drugs, contributing for acquiring relevant information from the system or turning the analytical method greener.

## 2. Degradation Products

The efficacy and safety of drugs are determined by toxicological and pharmacological profiles and adverse side effects due to the dosage and impurities [37,38,39]. According to the International Council for Harmonization and Technical Requirements for Pharmaceuticals for Humans Use (ICH), a drug impurity is any component that is not a chemical entity defined as an active pharmaceutical ingredient or excipient [40]. The impurities can be classified regarding their origin: inorganic impurities (reagents, ligands and catalysts, heavy metals or other residual metals, and inorganic salts), organic impurities (starting materials, by-products, intermediates, degradation products, reactants, ligands, and catalysts), and solvents (organic and inorganic liquids used in preparation of solutions or in the synthesis of a new drug substance). Therefore, any extra material present in the drug, even if it does not have pharmacological activity, is considered an impurity [39]. Although the term “impurity” is commonly assigned as synonymous of degradation products, it is worth highlighting that these compounds belong to a subgroup inside the impurity definition [41]. The United States Pharmacopoeia adopts the term “Related Compounds” for the main degradation products and impurities from synthesis.

The yielding of degradation products depends of several variables, chemical stability being the most important one. The degradation of APIs involves the formation or breaking of covalent bonds in chemical processes such as oxidation, reduction, thermolysis, and hydrolysis reactions. These processes can usually be accelerated when the drug is exposed to light, high temperatures, acidic or basic conditions, humidity, oxidative environment, incompatible excipients, and even due to its contact with packaging during its shelf-life [41].

### 2.1. The Generation of Degradation Products

Stability of API is a critical parameter in the development of a drug product, which should be considered in the formulation, analytical methods, package, storage, shelf life determination, safety, and toxicological studies [42,43].

The degradation of an API can result in the loss of effectiveness and can also lead to adverse effects due to degradation products [44]. Therefore, understating the processes that contribute to generation of degradation products is extremely important to create strategies aimed at the prevention and/or minimization of the API’s degradation.

The oxidative degradation is one of the leading causes of drugs degradation, once it involves the removal of an electropositive atom, radical, electron, or the addiction of an electronegative atom or radical. The major part of API’s oxidation occurs slowly due to the action of molecular oxygen, and some procedures used during manufacturing and storage are employed to stabilize the API in the product. For that, it is necessary to know the variables that increase the extension of oxidation. One form of preventing the oxidation process is to substitute oxygen inside pharmaceutical recipients by nitrogen or dioxide carbon. The contact of drug with metal ions, which can catalyze the oxidation, should be also avoided, as well as high storage temperatures [45].

Temperature is another variable that has significant influence on degradation and is often used in forced degradation studies. The same product can present different shelf lives depending on how and where it is stored. For example, countries in which equatorial climate predominates have higher average temperature than the ones with tropical climate, and this difference promotes different degradation conditions and, consequently, different shelf lives [46].

Several pharmaceutical drugs have low stability in aqueous medium and must be evaluated under hydrolysis conditions. First, to evaluate the hydrolysis of an API, it is necessary to perform tests in a wide range of pH (solution or suspension) once the hydrogen and hydroxyl ions are able to influence the degradation ratio [47,48,49]. Then, hydrolytic forced degradation studies are performed by submitting the API to acid, basic, and neutral conditions, in a fashion that the experimental variables have to be adapted if it is observed high degradation of API, in order to avoid the formation of secondary degradation products [48].

Photostability studies should also be performed to demonstrate the extension of reactions when the APIs are exposed to light. The photolytic reactions are caused when the drug absorbs the ultraviolet/visible (UV-Vis) light (wavelength 300 to 800 nm), which promote the molecule to an excited state and can increase its reactivity in some sites of the molecule. The UV-Vis radiation also can lead to cleavage of chemical bonds, yielding new molecules. The extension of photodegradation is dependent of the wavelength of the incident radiation and the absorptivity of the molecule. In other words, this process depends of the presence of specific functional groups [50].

Nonetheless, it is worth mentioning that even when an API is shown to be chemically stable in stress tests, the stress conditions can degrade this API when excipients are present.

### 2.2. Forced Degradation Studies

Since the release of the first guidelines, massive changes to the definition of quality in pharmaceutical drugs have taken place, and several countries are extending the requirements of regulatory agencies to generic drugs and already commercialized products [51]. Forced degradation studies, also called “stress tests,” have been used in the pharmaceutical industry for a long time [50], but the International Conference on Harmonization (ICH) only issued the formal request Q1A with a guideline “Stability Testing of New Drug Substance and Products” in 1993 [52]. In general terms, forced degradation studies are processes that involve the degradation of drugs under extreme conditions to accelerate the yielding of degradation products. The information obtained from these studies are usually used to determine the chemical stability, pathways of degradation, to identify the degradation products, conditions of storage, self-life, excipient compatibility, and also allow the development of selective analytical methods [52,53,54].

Today, the control of impurities has been established by ICH Q3A and Q3B guidelines, which are addressed for registration applications about the content and qualification of impurities classified as degradation products, which are observed during manufacturing or stability studies of the new drug product. Furthermore, the registration application should present a validated analytical procedure suitable for the detection and quantification of degradation products, which should include or evidence the method’s specificity for specified and unspecified degradation products according to ICH Q2A and Q2B guidelines for analytical validation. When the impurities are available in the validation method phase, the discriminatory capacity of drug and impurities is validated through spiking drug substance with levels of impurities. On the other hand, if impurity or degradation product standards are unavailable, the drug substance should be submitted to stress conditions (light, heat, humidity, acid/base hydrolysis, and oxidation). Therefore, in general, the forced degradation studies are performed in the developing stability-indicating method, and the method validation should take into account the chromatographic separation of the degradation products.

Several works in the literature deal with studies of forced degradation and stability as synonymous, but it is worth highlighting that there are some differences between them. Stability studies consist of submitting the pharmaceutical drug in milder conditions over a long period (months or years) and, besides determining some degradation products, allow the establishment of the product’s shelf life. Forced degradation studies are often performed by exposing the API or the product in drastic conditions for some hours or days. These extreme conditions are able to provide, as a general rule, substantial degradation of the API, usually from 10 to 30%. The set of whole degradation products found in every degradation condition composes a “potential” degradation profile. If just few degradation products are found, the degradation profile is then denominated as “real degradation profile.” The method to evaluate the degradation products should be selective and developed considering the occurrence of every degradation product [55].

The forced degradation studies are critical in the development of drug products and aims the following points:

- To obtain the potential degradation potential of an API or drug product;
- To discover the degradation mechanism, such as hydrolysis, thermolysis, oxidation, photolysis, etc.;
- To elucidate the molecular structure of degradation product;
- To solve problems regarded to the API stability;
- To identify the conditions where the API or the drug product are more susceptible to degradation in order to ensure the quality of the final product, bringing to pharmaceutical industry enough knowledge for development, packaging, manufacture, manipulation, and storage;
- To obtain more stable formulations;
- To develop analytical methods that can be used to quantify the API without interference of its degradation products and to quantify these degradation products [48,56,57].

The degradation products are commonly analyzed by high-performance liquid chromatography (HPLC) coupled with ultraviolet/visible (UV-Vis) and/or mass spectrometric (MS) detectors. UV-Vis detectors are able to provide only information related to chromophores groups, but they are excellent for quantification. MS detectors are not robust as UV-Vis detectors for quantification, but MS presents high sensitivity (traces level) and gives important data to characterize the degradation products through fragmentation profile, accurate mass (for detectors of High Resolution such as Q-ToF, Orbitrap, and Fourier-transform ion cyclotron resonance (FT-ICR)), as well as information about the origin of fragments using multiple stage (MS^n^) and neutral loss scan. When more information is necessary to elucidate a chemical structure, the nuclear magnetic resonance (NMR) technique is required. NMR presents low sensitivity, but it is able to resolve conformational, structural, and optical isomers. All these techniques generate a great amount of data, and the manual data mining is very time and money consuming. In this context, chemometric tools can present a way to organize and pre-process data, optimize parameters of HPLC, MS, and NMR techniques, obtain the maximum knowledge about them, and clarify a lot of useful information [51,58,59].

### 2.3. Strategies to Select the Degradation Conditions

Forced degradation studies are performed in batches with solutions at different pHs, in the presence of hydrogen peroxide, UV-Vis radiation, metallic cations (Fe^3+^ and Cu^2+^), and high temperatures [48].

Usually, the influence of pH is evaluated using 0.1 mol L^−1^ of HCl or NaOH [48]. The degradation by radiation is performed under UV-Vis light, which should not be lesser than 1.2 million of lux per hour and a power of 200 Wh m^−2^ [60]. For oxidant condition, the literature recommends using hydrogen peroxide (H_2_O_2_) in concentration from 0.1% to 3.0% at room temperature (25 °C). The evaluation of temperature is usually performed between 40 to 80 °C, but it could be higher for recalcitrant APIs. Other additional variables can be taken into consideration in the global stability studies of an API or the final product, such as humidity and microbiological stability [22,57,61,62].

According to ICH, in “Expert Committee on Specifications for Pharmaceutical Preparations” document, the recommended degradation should be between 10 to 30% of the API. This degradation range commonly allows for the evaluation of the main degradation products, avoiding the yielding of secondary degradation products [63]. In Brazil, the regulatory agency ANVISA recommends not less than 10% of degradation of API, and a technical justification is needed in the case where such degradation is not obtained [64].

It is worth highlighting that the cited conditions for forced degradation studies are just initial attempts, and the ideal condition could be more extreme or mild, depending of the chemical recalcitrance of the API. Table 1 summarizes degradation conditions of some papers that performed forced degradation studies.

### 2.4. Acceptable Limits of Impurities

After obtaining the degradation profile, a critical analysis should be performed to verify the purity of the chromatographic band of the API and to evaluate the variables that can promote degradation of the API. The degradation products are analyzed according to their amount in relation to the API in the final product, after the regular stability time (without any stress condition). The evaluation considers the maximum amount of API administered per day, and the limit of degradation products are expressed as a percentage (or mass) relative to the API. The amount of degradation products defines if it is necessary to perform notification, identification, or qualification [40,57,77]. Table 2 shows the acceptance criterion used by ICH, FDA, and ANVISA for the amount of impurities found in relation of a daily administrated API. The acceptance criteria have the following meaning:

- Reporting threshold: A limit of impurity that is not necessary to be reported.
- Identification threshold: A limit of impurity does not need to be structurally identified.
- Qualification threshold: The maximum amount of impurity that is not necessary to be qualified. Being “qualified” is the process of acquisition and evaluation of data that establishes biological security of an impurity or a degradation profile at the specified levels [40].

## 3. Applications of Chemometric Tools in Forced Degradation Studies

### 3.1. Design of Experiment (DoE)

In every area is important to know how variables act on the system. In general, processes aim to enhance the quality of the final product, taking into account the minimization of cost and time. To achieve these goals, it is necessary to perform the optimization of variables of the system to gain knowledge about the behavior of variables in order to determine the influence of each variable [78,79]. The optimization of variables in a system is more commonly performed using one-variable-at-a-time approach (OVAT), where one variable, or also called factor, is changed at a time, causing a change in the monitored response. However, this univariate approach does not consider the interactions between variables, and therefore, it does not ensure the discovery of the optimum point in an optimization process [80]. The design of experiments arises as an alternative multivariate approach for studying the behavior of a system [81]. In this approach, the factors are simultaneously evaluated, and the experiments are performed in an organized way in order to acquire information about all the system performing a minimum number of experiments [82,83].

Some terms in DoE must to be clear for better understanding, as variables, levels, and responses. Variables or factors are independent experimental inputs capable of changing the responses of the system. Such factors are temperature, pH, irradiation time, reaction time, concentration of reactants, and so on. It is worth reiterating that variables can be changed independently of each other, but the response is dependent of synergism between them [84].

Levels are different values that a variable can assume within experimental domain. The variable temperature in an optimization process, for example, can be studied at three levels: at 30, 50 and 70 °C.

Responses or independent variables are the monitored parameters. Typical responses are cost, time of analysis, resolution between chromatographic peaks, percentage of API degradation, etc.

**Figure 1:**
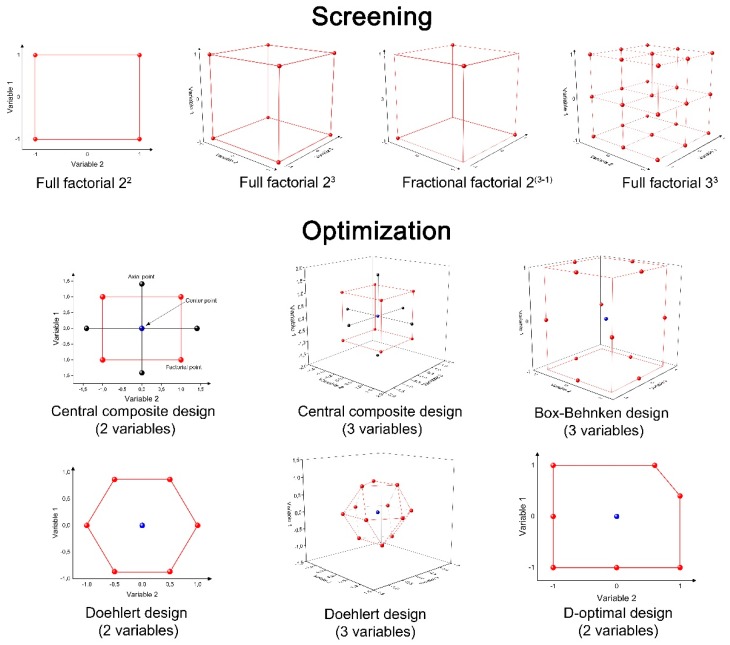
Experimental domain of the most common experimental designs.

The values studied for each variable are coded in levels as high (+1), central (0), low (−1), and other levels, which depend on the design. This codification normalizes the independent variables, avoiding any wrong interpretation of data. The processes involved in DoE allow it to fit the empirical data to a function, creating a linear or quadratic model and considering the interactions between variables of the system [85]. Figure 1 shows the experimental domain of the most common experimental designs for screening and optimization steps.

In sum, the DoE presents the following advantages:

- Determining how many experiments are necessary to achieve the goal;
- Reducing the number of experiments;
- Observing the synergic and antagonist interactions between variables;
- Allowing for the possibility to create mathematical models and surface response to describe the behavior of the variables and to predict the system’s response within an experimental domain;
- Decreasing the time, costs, and generation of lesser amounts of chemical waste, which contributes for the green chemistry principles [79].

In the context of forced degradation studies, the DoE has been mainly used for the development and optimization of chromatographic methods and for multivariate evaluation of stress conditions.

The use of DoE in the development and optimization of chromatographic conditions is not exclusive for forced degradation studies; instead, its application has spread to several fields that use chromatography as a tool [86,87,88]. Krishna et al. [89] performed forced degradation studies of eberconazole nitrate (EBZ) submitting it to hydrolytic (acid, basic, and neutral), thermal, oxidative, and photolytic degradation. In this work, a full factorial 3^3^ design was used to identify the best conditions of the mobile phase for drug analysis. As is already well known in chromatography, the organic modifier in the mobile phase (methanol in this case), pH (10 mM potassium dihydrogen orthophosphate), and ion pair agent (tetra butyl ammonium hydroxide, TBAH) are important variables and alter the capacity factor (k) of the mobile phase. These variables were evaluated in three levels (−1, 0, and +1) following a full factorial design with 27 experiments (3^3^ Full Factorial). Table 3 presents the real value of variables, and Table 4 shows the 27 different experiments.

The ranges studied in design were selected according to previous studies and considered the physicochemical properties of EZB. Other chromatographic parameters such as column dimensions, flow rate, injection volume, wavelength for detection, as well as the procedure performed in each degradation condition, can be found in reference [89].

As a result, a Pareto chart of standardized effects showed the quantification of each variable on the capacity factor, where organic phase and TBAH presented the higher influence on the response. Both linear and quadratic regressions showed no significance for pH inside its range of variation. The results of experimental design also allowed the authors to create contour plots, and they emphasized the usefulness of studying the interaction effects of variables on capacity factor. It was observed through contour plots that, by increasing concentration of TBAH, the capacity factor of EBZ was increased, and the same behavior occurred when the organic modifier decreased. Furthermore, pH did not affect the capacity factor in the investigated experimental domain. At the end, the optimum conditions (pH 2.8, 10 mM TBAH, and methanol 25% (*v*/*v*)) made it possible to find a capacity factor equal to 2.06.

Table 5 shows some papers that used the experiment design to optimize the chromatographic conditions to analyze the degradation products yielded in forced degradation studies.

In the papers presented in Table 5, the DoEs were used to evaluate the chromatographic parameters in order to obtain the best chromatographic method. The meaning of the best chromatographic method depends of the intention of the analyst—better resolution for the API, higher number of peaks in order to detect all degradation compounds, cost-and-time saving methods, etc.

Another purpose for forced degradation studies found by Sonawane and Gide [101] was the application of experimental design for the optimization of forced degradation of luliconazole (LCZ), 4-(2,4-dichlorophenyl)-1,3-dithiolan-2-ylidene-1-imidazolylacetonitrile), which is recommended for the treatment of fungal infections. The LCZ was submitted to acidic (HCl), alkaline (NaOH), oxidative (H_2_O_2_), thermolytic (under reflux), and photolytic (direct sunlight) stress conditions, and a full factorial design was chosen to identify the conditions to obtain a degradation of this API between 10 and 20%. The 2^3^ factorial design for acid and alkaline conditions took into account the variables concentration of the degradant agent (x_1_), temperature (x_2_), and time of exposure (x_3_) to achieve the desired degradation. The variable temperature was not included in oxidative degradation, and the design became a 2^2^ factorial design. The same design was performed to dry heat and wet heat degradation, but including the variable temperature and discarding the variable concentration. For photolytic degradation, LCZ powder was exposed to direct sunlight for 48 h and compared with control in dark, but DoE was not applied. The level of the variables for each stress condition is presented at Table 6. The 2^3^ factorial design was performed in a total of eight experiments, and the 2^2^ factorial in a total of four experiments for each degradation (oxidative, dry heat, and wet heat) by design. Table 7 shows the experiments and the obtained results by liquid chromatography.

The dry and wet heat degradation did not present any degradation of luliconazole, but photolytic degradation obtained 8%. Concerning acid, alkali and oxidative conditions, the degradation ranges were 0–41%, 0–43%, and 0–100%, respectively. Multivariate regressions were performed on the results for each degradation (acid, alkali, and oxidative) in order to obtain the regression models (equations) for the studied experimental domain. These regression models are used to predict suitable conditions to achieve the desired percentage of degradation. These conditions provided degradation of 11%, therefore, a relative error equal to 9%. More details about the equations in each degradation condition as well as surface response created to better visualization of the results can be found in the reference [101]. The DoE in this work allowed the authors to gain knowledge about stability of LCZ, presenting the degradation condition where LCZ is more susceptible to undergo degradation and indicating the variables that present higher influence on the degradation of LCZ. Finally, the chemometrics tools aid to predict the values of variables to obtain the desired degradation.

Another example was presented by Kurmi et al. [102]. that used DoE to develop the stability-indicating method and also found the stress conditions for forced degradation of furosemide in the range of 20–30%.

Despite the fact that DoE is a very interesting tool to find the most suitable conditions in the degradation studies and avoiding the generation of secondary degradation products, there are few papers presenting such approach.

### 3.2. About Fusion QbD^®^

As mentioned previously, forced degradation studies are performed in the development stability-indicating method phase. DoE is extremely useful to build a set of screening, optimization and robustness experiments. In this context, some HPLC method development software platforms are commercially available to automatically perform the experimental design. This software, such as Fusion QbD, uses concepts of experimental design and creates a sequence of experiments considering all relevant chromatographic parameters. It is possible to build, for example, a set of screening experiments considering more than one type of chromatography columns, multi-solvents, and other chromatographic variables. After the creation of a set of methods, guided by the DoE principles, and after running the sequence of experiments, the software generates mathematical models and makes predictions to find the better chromatographic method. As Fusion QbD is integrated with the chromatography system, all functions of HPLC are explored, and it allows users to reach maximum efficiency and speed in the method developing process [103]. Others specialized software is also used to create basic designs, such as Origin [104], Matlab [105], Minitab [106], Design-Expert [107], and Statistica [108].

### 3.3. Principal Component Analysis (PCA)

Principal component analysis (PCA) is one of the most used chemometric tools for data exploration through the reduction of a system’s dimensionality [23,109,110]. This technique allows the user to establish the numerical adjustment of a linear model for describing the central relationships among process variables [111]. The PCA aims mainly to extract the most useful information from data. Besides, this chemometric tool helps simplify the description of the data for the analysis of variables [112].

**Figure 2:**
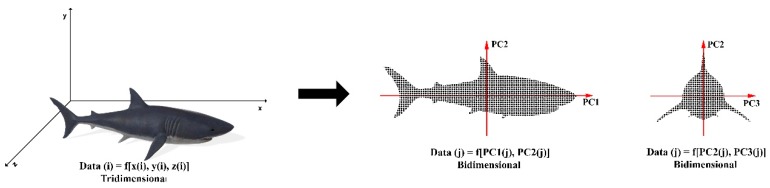
Representation of principal component analysis (PCA). Original data at left side, PC1 × PC2 in the middle and PC2 × PC3 at right side.

The use of PCA enables the user to represent objects with new variables that are linear combinations of the original variables. These linear combinations, denominated principal components (PCs), are calculated considering directions of maximum variance, in a fashion that they may also be perpendicular to each other [23]. The first PC describes the maximum variance of the sample. The second PC describes the most considerable variability that the first one was not able to describe. The directions of the most dispersed samples are generally described in the first PC, since it corresponds to the vector with more information about the linear combinations of the original variables [113]. Figure 2 presents a graphical representation of PCA, where the axes are changed in order to maximize the explained variance using a smaller number of dimensions.

In the literature, three papers were found involving PCA associated with degradation products of pharmaceutical drugs. Two of them will be discussed in the next paragraphs, and the other one will be discussed later, in the MCR-ALS context.

Tôrres et al. [114] performed accelerated degradation studies of captopril and applied Multivariate Statistical Process Control (MSPC) for monitoring and identifying any changes in samples in order to guarantee the product quality. The details of all procedure data treatment can be found in reference [114]. The captopril stability was evaluated leaving 24 blisters of tablets of the same batch in a climatic chamber at 40 ± 2 °C and 75 ± 5% of relative humidity. One blister per week was analyzed by liquid chromatography, for six months, totalizing 24 chromatograms. In order to build the process control chart, a sample set of Captopril was used under normal operation conditions in the calibration (training stage), and in the validation stage, samples were used under normal operation conditions, as were samples presenting expired shelf life. Hotelling’s T^2^ statistic and Square Prediction Error (SPE) were used for sample monitoring. PCA is a useful tool in the Hotelling’s T^2^ statistic, since it reduces the number of variables to be monitored, changing the original variables by the scores in the PCA, without significant information loss from dataset. The PCA along with the multivariate control charts contributes to identify possible failures and changes early in the process, making this method useful to ensure the quality control of product [114]. The same authors also performed a similar work using the mid (MIR) and near (NIR) infrared techniques [115].

Skibinski et al. [66] performed forced degradation of toloxatone, which is a pharmaceutical drug used as an antidepressant. These studies were carried out in basic (0.01 M NaOH), acidic (1 M HCl), neutral (water), photo UV-Vis, photo UVC, and oxidative (0.01% H_2_O_2_) degradation conditions. The samples (including the control solution) were evaluated in a LCMS (ToF) totalizing 21 chromatographic profiles. The stress conditions provided eight unique degradation products of toloxatone [66].

After aligning of chromatographic profiles, PCA analysis showed a visible grouping of the stressed samples. The author noticed that stressed basic samples gave rise to a separated cluster from other stressed samples in the scores analysis obtained from PCA, while neutral and acidic samples were close to the control samples. On the other hand, it was possible to separate in groups the samples carried out under photo UV-VIS, photo UVC, and oxidation conditions. The first three components of PCA model were able to explain almost 71% of the total variance. This work shows that PCA analysis can be used as a tool to characterize the chromatographic profiles.

### 3.4. Partial Least Squares (PLS)

Partial least squares (PLS) regression is a multivariate regression technique, the most important one in the chemometrics. It is used to stablish quantitative relationships between a vector of information (UV-Vis, Raman, NIR, MID-IR, NMR spectra or chromatogram, diffractogram, etc.) and properties to be quantified (concentration of an analyte, crystalline phase of API, etc.) [116,117,118,119].

As example, the concentrations of an analyte in calibration samples are organized in a vector y, and the chemical data (spectra) are organized in a matrix X. In the classic multivariate regression, the regression coefficient **b** is found by **b** = **y ×** X^+^, where X^+^ is the pseudoinverse of X. The regression equation (model) can be written in the matrix form as **y** = **b ×** X. However, there is some issues related to the use of classical multivariate regression, such as the need of high number of samples and the problem of the correlation among the variables in the matrix X. Then, in a similar way as PCA, PLS calculations simultaneously decomposes X and **y** in order to maximize the correlation among the scores of X and **y**. After defining coefficients **b**, it can be applied to determine the concentration in external samples [120].

Some algorithms have been proposed to perform PLS, and the most common are PLS1 and PLS2, for one response and for multiple responses, respectively. Although PLS2 is used for multiple responses, it is recommended only in the cases where there is high correlation among the responses [121].

Recently, Sayed et al. [122] developed a stability-indicating method using PLS to determine mometasone furoate (MF) pure or in pharmaceutical formulation in the presence of its degradation products. The forced degradation was performed only in basic conditions once other previous works have demonstrated its susceptibility in undergoing alkaline hydrolysis. The multilevel multifactor experimental design was applied to prepare mixtures of calibration set constituted by 14 samples, which were scanned over the range of 220–350 nm. The UV spectra of 11 different mixtures of MF and its degradation products were used to predict the concentration of MF. The PLS model applied in the determination of MF presented good results, obtaining in calibration set mean recovery of 100.2% and RMSEC 0.002% meanwhile validation set presented mean recovery of 97.24% and RMSEP 0.04%. The recoveries in pharmaceutical samples were also satisfactory (98.47–102.66%), demonstrating no interference from excipients or alkaline degradation products in the quantification and the power of PLS method for quantification of MF [122]. Besides, in this same work, a new TLC densitometric method and the chemometric tools CLS and PCR were found, which were applied to develop quantification models for the MF in pharmaceutical samples.

Attia et al. [123] also developed spectrometric methods for determination of cefoxitin-sodium in the presence of its alkaline degradation product using different chemometric tools. PLS was applied to quantify cefoxitin-sodium in pharmaceutical sample. To obtain degradation product, the basic forced degradation was performed using NaOH 0.1 M for 10 min, which was neutralized with HCl 0.1 M. More details about the procedure to prepare the working solution are in reference [123]. The PLS model was built considering 13 mixtures denominated calibration set and 12 mixtures as a validation set obtained through experimental design. The number of factors was optimized through cross-validation method, as performed in reference [122]. The genetic algorithm (GA) was coupled with PLS to improve the prediction capability of models eliminating variables without information. In fact, the efficiency of the calibration of GA-PLS was better than only PLS, given lower RMSEC and RMSEP values for GA-PLS. The analysis of cefoxitin-sodium in presence of degradation products and in the pharmaceutical sample presented mean recovery of 100.54% and 99.86 ± 1.347%, respectively, using GA-PLS. The proposed method presented no significant difference compared to the standard method. Different chemometric tools were proposed and all of them showed a solvent reduction and sample consumption, making the methods greener. Table 8 present papers found in the literature that use in some moment the PLS tool in forced degradation studies of pharmaceutical products.

### 3.5. Multivariate Curve Resolution (MCR)

Multivariate curve resolution (MCR) has been widely used to analyze several types of data in different application fields [137,138,139]. MCR constitutes a bilinear model based on the classical least squares (CLS) that decomposes data matrix into two submatrices, which have chemical information of the compounds involved in the system [137,139,140,141].

This approach is also known to be spectral unmixing tool once it allows mathematically solving analyte signals of a complex mixture where they are overlapped in one or more dimensions of data, as chromatograms and spectra of analyte in the presence of interferents in analysis without resolution. MCR aims to differentiate the individual contributions of components of a mixture providing the pure signals (spectra) and the proportions of analytes through concentration profile [138,139,142]. MCR comes from the Beer’s law, where concentration is proportional to the absorbance. In this way, a spectral data set can be deconvoluted in the pure spectra from the analytes and their relative concentration. The general equation for MCR is X = C × S^t^, where the spectral matrix X is deconvoluted in the concentration matrix and the pure spectra matrix.

Most papers related to forced degradation studies and MCR-ALS aimed for the evaluation of photodegradation. Except for basic hydrolysis condition, other degradation conditions were not found in the literature.

Marín-García et al. [143] investigated photodegradation of tamoxifen in aqueous medium using Multivariate Curve Resolution-Alternating Least Squares (MCR-ALS). The photodegradation experiments were conducted at 35 °C in a cabinet equipped with light at two different irradiation power conditions (400 and 765 W/m^2^) according to ICH requirements. To monitor the photodegradation of tamoxifen, the UV-VIS spectra were collected from 0 to 160 min for irradiation power 400 W/m^2^, and from 0 to 120 min for 765 W/m^2^. The UV spectra allowed to obtain the evolution of the photodegradation process. MCR-ALS analysis of the UV data allowed to observe the estimation of the kinect profiles for the possible presence of at least four species, three of them being degradation products. Besides, it was possible to obtain the relative concentration of each specie along time.

During photodegradation some molecules cannot be detected by UV-Vis due to the loss of chromophore groups. The authors overcame this situation using a LC-DAD-MS technique to obtain deeper knowledge about species formed in photodegradation. In this case, MCR-ALS analysis provides the C and S matrixes that contain, respectively, the elution profile and pure UV-VIS or MS spectra for each substance. These matrixes showed a new component, which represents a fourth degradation product. This new specie was not observed in the UV-VIS monitoring, it rises during photodegradation but disappears at the end of the process. Furthermore, the authors elucidated the degradation product structures. This work shows MCR-ALS’s ability to monitor and solve mixtures of degradation products formed during photodegradation process [143].

Another work reported in the literature was conducted by Feng et. al. [144], which investigated the basic degradation for paracetamol using two-way dimensional UV-Vis associated to MCR-ALS. Forced degradation was performed using a quartz cell where paracetamol and NaOH solutions were added, and the UV-VIS spectra were collected from 1 s to 24 h. Initially, a PCA was applied on UV-VIS data, and it suggested the existence of four components. Later, the concentration profiles were obtained from evolving factor analysis (EFA), and it confirmed the number of chemical components involved in degradation reaction. In the MCR-ALS deconvolution, it was applied to the constraints non-negativity for spectral and concentration profiles and unimodality for the concentration profile. Through the concentration profile and spectra profile plots, it was possible to perform a critical analysis of the formation and consumption of the species during alkaline degradation. It was possible to observe that there were a reactant, a degradation product, and two intermediates. The authors compared the results with HPLC analysis, which proved the existence of two intermediates, and the concentration profile were in agreement with the one recovered by MCR-ALS using UV-Vis. Besides, the authors also proposed a degradation pathway in alkaline media. The use of MCR-ALS in forced degradation studies allowed to verify the drug stability and kinect of degradation of paracetamol [144]. Other papers regarding forced degradation studies and MCR-ALS are presented in Table 9.

### 3.6. Artificial Neural Network (ANN)

Artificial neural networks (ANNs) are powerful chemometric tools based on artificial intelligence. They can model nonlinear data through learning processes in a similar way to the human brain [36,152]. ANN models are able to map the input data in a set of appropriate outputs following a “learning by examples.” In other words, the structure of data is learned through training algorithms [153].

To the best of our knowledge, two works regarding to forced degradation studies and artificial neural network are reported in the literature, and only one of them uses ANNs as the main tool [123,154].

Golubović et al. [154] used ANNs to develop quantitative structure-retention relationships (QSRRs) model to optimize isocratic RP-HPLC method of candesartan cilexetil in the presence of seven degradation products obtained from acid, alkaline, neutral hydrolysis, photolysis, and oxidation conditions. QSRRs is able to relate chromatographic retention parameters and molecular structure, and it becomes a valuable tool to the prediction of chromatographic behavior and separation of complex mixtures.

Initially, to investigate the variables that could influence the chromatographic behavior, a 2^5–1^ fractional factorial design was performed. The following variables were included in the design: percentage of acetonitrile in the mobile phase, buffer pH and ionic strength, temperature of the column, and flow rate of the mobile phase. All variables showed to be significant and, therefore, were considered as inputs in the ANN modeling, except flow rate, which was maintained as a constant.

The molecular structure is an essential variable in QSRR model and is encoded by descriptors. Roughly, molecular descriptors are obtained by logic and mathematical procedures that transform chemical information in a useful number of some standardized experiments. The selection of molecular descriptors was based on intermolecular interactions suggested by theory of liquid chromatography. In the ANN modeling it were included the descriptors which present low correlation between them, such as polarizability, H-donor sites, H-acceptor sites, and octanol/water distribution coefficient.

It was used a multi-layer feedforward, the most common ANNs, constituted by one input layer (descriptors and significant chromatographic variables), number of hidden neurons connected to both input and output neurons (retention factor). In the network training stage, the overall agreement between computed and target output for a set training is maximized. In order to avoid overfitting, the predictive power of network was evaluated using a validation set. Both training and validation sets were defined through a Box-Behnken design, varying from −1 to +1 level. A total of 344 cases for ANN optimization were obtained, which were divided into 280 cases for the training set, 32 for external validation, and 32 to validation set. For training, validation, and external validation data sets, coefficients of determination (R^2^) were obtained between experimental and predicted retention factor (K_exp_ and K_ANN_ respectively) equal to 0.9993, 0.9969 and 0.9956, respectively. Therefore, high R^2^ and low RSME values demonstrate an excellent predictive ability of model and non-occurrence of overfitting during the training process.

This kind of mathematical model is an important tool in forced degradation studies since degradation products derive from the API and, therefore, are chemically similar. The creation of models able to predict the behavior of active substance and all degradation products contribute to defining the optimal chromatographic conditions during the optimization process [154].

## 4. Conclusions

Chemometric tools can bring considerable gains in forced degradation studies. DoE is the most used chemometric tool in such studies, especially in the development of suitable chromatographic methods to monitor the API. However, the application of DoE directly in stress experiments is also promising, as it is possible to quantify the individual effect of stress variables as well as the synergy between them, simulating what may occur in real life. The other widely used tool is PLS, since its use allows the quantification of the API directly in UV-Vis spectrophotometry analyzes, since it performs multivariate quantification, which makes possible quantification of species without resolution.

The PCA technique is not applied in these studies since it is an exploratory method, and its application is more related to process monitoring and classification methods for raw material identification.

The other tools, despite being very useful in such studies, are more complex, and their application is limited for non-chemometricians.

## Figures and Tables

**Table 1:** Degradation conditions for pharmaceutical drugs in forced degradation studies.

API: Year	Acid	Base	Neutral	Thermolysis	Oxidation	Photolysis
Zidovudine: 2017 [65]	2 M HCl	2 M NaOH	-	Acid/base at 80 °C for 72 h	10% H_2_O_2_ at room temperature for 10 h	1.2 × 10^6^ lx × h of fluorescent light and 200 W h/m^2^ UV light
Toloxatone: 2018 [66]	1 M HCl	0.01 M NaOH	H_2_O	All hydrolysis at 80 °C for 2 h	0.01% H_2_O_2_ at room temperature for 2 h	2700 kJ/m^2^/h of UV-VIS and UVC 7.5 W/m^2^
Amlodipine: 2015 [67]	1 M HCl at 80 °C for 30 min	1 M NaOH at 80 °C for 1 h	H_2_O at 80 °C for 2 h	50 °C for 48 h	15% H_2_O_2_ at room temperature for 48 h	1.2 × 10^6^ lx × h of fluorescent light and 200 Wh/m^2^ UV-A light for 14 days
Acebutolol: 2018 [68]	1 M HCl	2 M HCl	H_2_O	All hydrolysis at 80 °C	3% H_2_O_2_ at 80 °C	Not less than 1.2 × 10^6^ lx × h and ultraviolet energy of not less than 200 W h/m^2^
Stevioside: 2018 [69]	0.1 M HCl/0.1 M H_3_PO_4_	0.1 M NaOH	H_2_O	All hydrolysis at 80 °C for 8 h	10% H_2_O_2_ at 25 °C for 72 h	UV_254nm_ lamp for 48 h
Pentoxifylline: 2013 [70]	2 M HCl at 70 °C for 4 h	2 M NaOH at 70 °C for 4 h	H_2_O at 70 °C for 4 h	Dry heat under at 105 °C for 4 h	30% H_2_O_2_ at 70 °C for 4 h	Sunlight for 8 h
Leflunomide: 2015 [71]	0.1–5 M at 85 °C for 8 h	0.1 M NaOH at 85 °C for 8 h	H_2_O at 85 °C for 8 h	50 °C for 30 days	30% H_2_O_2_ at room temperature for 24 h	UV and white light for 14 days
Actarit: 2014 [72]	0.1 M HCl at 70 °C for 24 h	0.1 M NaOH at 70 °C for 24 h	H_2_O at 70 °C for 14 days	Dry heat at 70 °C for 14 days	3% H_2_O_2_ for 14 days	UV light
Nicardipine: 2014 [73]	1 M HCl at 60 °C for 1 h	0.1–0.5 M NaOH at 50–80 °C for 1 h	-	-	5% H_2_O_2_ at 30–50 °C for 1 h	UV_254–365nm_ light at room temperature
Clopidogrel bisulfate: 2010 [74]	1 M HCl	1 M NaOH	-	All hydrolysis at 80 °C for 1 h	5% H_2_O_2_	-
Biapenem: 2009 [75]	pH from 2.5 to 7.5 at 80 °C for 40 min			From room temperature to 100 °C in pH 3.5	-	-
Irbesartan: 2010 [76]	1 M HCl at 80 °C for 24 h	2 M NaOH at 80 °C for 48 h	H_2_O at 80 °C for 48 h	50 °C	30% H_2_O_2_ at room temperature for 2 days	8500 lx fluorescent and 0.05 W/m^2^ UV light

**Table 2:** Thresholds for degradation products.

	Maximum Daily Dose	Threshold
**Reporting Threshold**	≤1 g	0.1%
	>1 g	0.05%
**Identification Threshold**	<1 mg	1.0% or 5 µg TDI, whichever is lower
	1 mg–10 mg	0.5% or 20 µg TDI, whichever is lower
	>10 mg–2 g	0.2% or 2 mg TDI, whichever is lower
	>2 g	0.10%
**Qualification Threshold**	<10 mg	1.0% or 50 µg TDI, whichever is lower
	10 mg–100 mg	0.5% or 200 µg TDI, whichever is lower
	>100 mg–2 g	0.2% or 3 mg TDI, whichever is lower
	>2 g	0.15%

**Table 3:** Real and coded values of variables considered in design of experiment.

Variable	Level (−1)	Level (0)	Level (+1)
TBHAH (mM)	5	7.5	10
pH	2.6	2.9	3.2
Organic phase (*v*/*v*)	20	25	30

**Table 4:** Conditions of experiments performed in full factorial 3^3^ design.

Experiment	x_1_	x_2_	x_3_	Experiment	x_1_	x_2_	x_3_	Experiment	x_1_	x_2_	x_3_
**1**	−1	−1	−1	**10**	−1	−1	0	**19**	−1	−1	1
**2**	0	−1	−1	**11**	0	−1	0	**20**	0	−1	1
**3**	1	−1	−1	**12**	1	−1	0	**21**	1	−1	1
**4**	−1	0	−1	**13**	−1	0	0	**22**	−1	0	1
**5**	0	0	−1	**14**	0	0	0	**23**	0	0	1
**6**	1	0	−1	**15**	1	0	0	**24**	1	0	1
**7**	−1	1	−1		−1	1	0	**25**	−1	1	1
**8**	0	1	−1	**17**	0	1	0	**26**	0	1	1
**9**	1	1	−1	**18**	1	1	0	**27**	1	1	1

**Table 5:** Design of experiments used in some papers to optimize chromatographic conditions for analyses of degradation products.

API	Design	Ref
Teriflunomide	Full factorial 3^3^	[90]
Simvastatin	Plackett Burman/Box-Behnken	[91]
Linagliptin	Full factorial	[92]
Ticagrelor	Fractional Factorial Resolution V/Central composite	[93]
Imatinib mesylate	Box Behnken	[94]
Fusidic acid	Taguchi/Central Composite	[95]
Cloxacillin	Plackett Burman	[96]
Vilazodone hydrochloride	Central composite experimental	[97]
Darifenacin hydrobromide	Central composite	[98]
Edaravone	Placket Burman/Box Behnken	[99]
Sofosbuvir and Ledipasvir	Box Behnken	[100]

**Table 6:** Real values of the variables used in the design of experiments.

Variable	High Level (+1)					Low Level (−1)				
	Acid	Basic	Oxid.	Dry Heat	Wet Heat	Acid	Basic	Oxid.	Dry Heat	Wet Heat
**Conc. (x_1_)/mol×L^−1^**	1	0.1	30%	-	-	0.1	0.01	3%	-	-
**Time (x_2_)/min**	75	30	24 h	360	120	15	10	2h	30	30
**Temperature (x_3_)/°C**	100	100	-	200	100	60	60	-	50	60

**Table 7:** Design of experiments with coded values and % of degradation of active pharmaceutical ingredient (API) for acid, basic, and oxidative conditions.

2^3^ Full Factorial Design						2^2^ Full Factorial Design			
Exp.	X_1_	X_2_	X_3_	Acid Condition	Basic Condition	Exp.	X_1_	X_2_	Oxidative Condition
**1**	−1	−1	−1	0%	0%	1	−1	−1	0%
**2**	+1	−1	−1	4%	3%	2	−1	+1	48%
**3**	−1	+1	−1	10%	8%	3	+1	−1	51%
**4**	+1	+1	−1	23%	11%	4	+1	+1	100%
**5**	−1	−1	+1	8%	19%				
**6**	+1	−1	+1	32%	26%				
**7**	−1	+1	+1	21%	38%				
**8**	+1	+1	+1	41%	43%				

**Table 8:** Works involving forced degradation studies and the partial least squares (PLS) tool.

Author	API	Forced Degradation Condition	Chemometric Tool	Year	Ref.
Attia et al.	Cefprozil	Basic hydrolysis	PLS; SRACLS	2016	[124]
Alamein et al.	Pimozide	Acid and basic hydrolysis	CLS; PCR; PLS	2015	[125]
Hegazy et al.	Linezolid	Acid and basic hydrolysis; oxidative	PLS; PCR; Parafac; N-PLS	2014	[126]
Hegazy et al.	Imidapril hydrochloride	Basic hydrolysis; oxidative	PCR; PLS	2014	[127]
Souza et al.	Captopril	Thermolysis	PLS	2012	[128]
Abou Al Alamein	Zafirlukast	Basic hydrolysis	PLS	2012	[129]
Naguib	Bisacodyl	Acid hydrolysis	PLSR; SRACLS	2011	[130]
Abdelwahab	Atenolol; Chlorthalidone	Acid and basic hydrolysis	PCR; PLS	2010	[131]
Wagieh et al.	Oxybutynin hydrochloride	Basic hydrolysis	PCR; PLS	2010	[132]
Moneeb	Rabeprazole sodium	Acid hydrolysis	CLS; PCR; PLS	2008	[133]
S Fayed et al.	Cilostazol	Acid hydrolysis	PLS; CRACLS	2007	[134]
Ragno et al.	Lacidipine	Photodegradation	PLS; PCR; MLRA	2006	[135]
Shehata et al.	Rofecoxib	Basic hydrolysis; photodegradation	PLS; CRACLS	2004	[136]

**Table 9:** Works involving forced degradation studies and the Multivariate Curve Resolution-Alternating Least Squares (MCR-ALS) tool.

Author	API	Forced Degradation Condition	Chemometric Tool	Year	Ref.
Gómez-Canela	5-Fluorouracil	Photodegradation	MCR-ALS	2017	[145]
Bērziņš et al.	Furazidin	Basic hydrolysis	HS-MCR-ALS	2016	[146]
Luca et al.	Amiloride	Photodegradation	MCR-ALS	2012	[147]
Sílvia Mas et al.	ketoprofen	Photodegradation	MCR-ALS; HSMCR	2011	[148]
Luca et al.	Nitrofurazone	Photodegradation	HS-MCR-ALS	2010	[149]
Javidnia et al.	Nitrendipine and felodipine	Photodegradation	MCR	2008	[150]
Shamsipur et al.	Nifedipine	Photodegradation	MCR	2003	[151]
